# AGE AND SITE DIFFERENCES IN PLANNED AND PERFORMED ACTIONS IN RESPONSE TO IDENTIFIED RISKS IN OLDER ADULTS

**DOI:** 10.1093/geroni/igac059.3012

**Published:** 2022-12-20

**Authors:** Deborah Finkel, Linda Johansson, Björn Westerlind, Ulrika Lindmark, Marie Ernsth-Bravell

**Affiliations:** Indiana University Southeast, New Albany, Indiana, United States; Jönköping University, Jönköping, Jonkopings Lan, Sweden; Linköping University, Linköping, Ostergotlands Lan, Sweden; Karlstad University, Karlstad, Varmlands Lan, Sweden; Jönköping University, Jönköping, Jonkopings Lan, Sweden

## Abstract

The Swedish health care system focuses on allowing older adults to “age in place”; however, that approach assumes that home health services are adequate to support health and prevent unnecessary decline. Data from the Senior Alert national quality register in Sweden were examined to compare the quality of care across care locations. First registration in Senior Alert was available for 2914 adults aged 57–109 (median age = 81): 3.6% dementia unit, 7.8% home health care, 4.4% rehabilitation unit, 62.8% hospital, 21.4% care home. There were significant differences across units in the number of identified risks in 4 categories: falls, malnutrition, oral health, and pressure ulcer. Individuals in rehabilitation units averaged 2.4 risks, individuals in dementia and care homes averaged 2.0 risks, and individuals in home health care and hospitals averaged 1.4 risks. For individuals with identified risks, the differences between planned and performed actions for each risk independently were greatest for those in home health care. Moreover, the correlation between total planned and performed actions in home health care was .79 for adults aged 65–80 years and .39 for adults aged 81 and over. The correlation did not differ across age for the other care units. Results suggest that individuals most in need of actions to address health risks (older adults in home health care) are least likely to have the actions performed. Training and support of workers responsible for home health care need to be improved if the “age in place” policy is to continue.

